# Efficient Gas Barrier and Antibacterial Properties of Poly(lactic acid) Nanocomposites: Functionalization with Phytic Acid–Cu(II) Loaded Layered Clay

**DOI:** 10.3390/ma13092033

**Published:** 2020-04-27

**Authors:** Yongzhen Lei, Long Mao, Jin Yao, Zhihan Li

**Affiliations:** 1 Key Laboratory of Advanced Packaging Materials and Technology of Hunan Province, Hunan University of Technology, Zhuzhou 412007, China; leiyz@hut.edu.cn (Y.L.); lizh@hut.edu.cn (Z.L.); 2 Fujian Provincial Key Laboratory of Functional Materials and Applications, Xiamen University of Technology, Xiamen 361024, China

**Keywords:** layered double hydroxides, phytic acid, antibacterial activity, polylactic acid, barrier properties

## Abstract

Poly(lactic acid) (PLA) represents one of the most promising and attractive bio-based polymers for green packaging. However, toughness, gas barrier and antibacterial properties of pure PLA films cannot compete with those of traditional petroleum-based active packaging plastics. To fill this gap, utilization of excellent chelating properties of phytic acid (PA), functionalized layered double hydroxides (LDHs@PA-Cu(II)) was firstly synthetized via facile deposition and chelation of one-step assembled PA-Cu(II) coordination compounds on the surface of layered clay. Furthermore, LDHs@PA-Cu(II)/PLA nanocomposites were prepared by blending LDHs@PA-Cu(II) and pure PLA via solution casting evaporation process. After adding only 1 wt % LDHs@PA-Cu(II), elongation at break and tensile strength increase by 53.0% and 18.9%, respectively, and the oxygen relative permeability decreases by 28.0%. Due to the strong interface interaction and heterogenous nucleation, the reinforcement effect of LDHs@PA-Cu(II) at low loadings is remarkable. Meanwhile, owing to the antibacterial activity of PA-Cu(II) coatings, the antibacterial rate (against Escherichia coli) of LDHs@PA-Cu(II) exceeds 99.99%. Furthermore, the corresponding LDHs@PA-Cu(II)/PLA nanocomposites also show outstanding antibacterial properties, which will be a promising candidate for active packaging application.

## 1. Introduction

In recent years, food safety concerns have given rise to the development and relevant applications of active packaging materials [1,2]. Active packaging can prolong the shelf life of food by improving the gas barrier, antibacterial and antioxidant properties of packaging materials [3,4]. Meanwhile, as a promising form of active packaging, antibacterial packaging is beneficial to food preservation, the reason is that active packaging can delay food spoilage through the deactivation or inhibiting the growth of various microorganisms [3]. Therefore, while trying to reduce the gas barrier properties of packaging materials, using appropriate antibacterial packaging technology is also an effective way to prolong the shelf life of food.

A majority of polymeric packaging materials are produced from non-renewable petroleum resources, and eventually become spontaneous and non-degradable waste [5]. It is strongly necessary to develop fully biodegradable polymeric packaging materials with excellent comprehensive performance to alleviate the dependence on petroleum-based polymer. Among all these biodegradable polymers, poly(lactic acid) (PLA) has attracted considerable attention in the recent decades due to its effective biodegradability and availability from renewable resources such as starch and corn [6]. PLA is considered as a valuable candidate to replace petroleum-based polymer in many fields (packaging, medicine and agriculture, etc.) because of its good optical, thermomechanical properties, and processability [3,7]. However, in the face of various packaging conditions, the gas barrier properties and antibacterial activity of pure PLA are difficult to meet the requirements of active packaging. 

PLA based naonocomposites with improved properties have been studied extensively. Recently, layered clay with low cost, non-toxicity and human compatibility has received increasing attention in the blending modification of PLA. Considered as a natural barrier material, layered clay has an impermeable layered structure, which can prolong the permeation pathway of gas molecules in the composite film [8,9,10]. Among the layered clay, layered double hydroxides (LDHs) have the advantages of versatility, homogeneous structure, and large specific surface area as well as designable chemical composition [11,12]. However, previous studies about the properties of LDHs/PLA nanocomposites were mostly confined to thermal and mechanical properties, and little on the gas barrier properties and antibacterial activity. Gonçalves reported the investigation of intercalated CaAl-LDHs/PLA nanocomposites [13]. It was revealed that the thermal stability and UV barrier properties of PLA nanocomposites were higher than those of pure PLA. Katiyar reported that LDHs/PLA nanocomposites were prepared by *in-situ* polymerization of lactide dimer intercalated within LDHs [14]. However, the molecular weight of PLA was significantly decreased in the presence of LDHs. According to previous reports, both intercalation and *in-situ* polymerization of LDHs can improve the dispersion of LDHs in PLA matrix, but they may be complex and unstable modification processes. Surface organic coating is a very effective and convenient modification method, which can improve the interfacial adhesion between LDHs and polymer matrix. In the process of surface modification, antibacterial substances can also be loaded on the surface of LDHs, so as to realize multifunction of LDHs. Our research group has been committed to the application of surface functionalized LDHs to various polymers matrix [15,16,17]. We have reported that tannic acid-Fe(III) coated LDHs was firstly synthetized via deposition and chelation of tannic acid with ferric iron [15]. The results showed that elongation at break and oxygen barrier properties increased by 47% and 21% respectively at a low surface functionalized LDHs loading of 1 wt % in poly(ε-caprolactone) matrix. It indicates that the application of surface functionalized LDHs has great research potential in polymer matrix. 

**Scheme 1:**
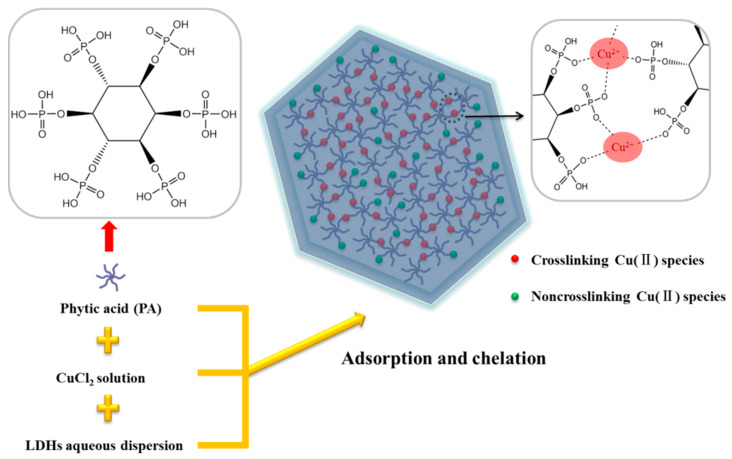
A schematic illustration of the synthesis for LDHs@PA-Cu(II).

Phytic acid (PA) is a natural substance extracted from legume seeds, cereal grains and beans [18]. It has attracted more and more attention in food additives, antibacterial agents and metal corrosion-resistant in recent years because of its unique properties such as non-toxicity, low cost, good biocompatibility and environmental protection [19]. More importantly, PA consists of six phosphates groups attached symmetrically to a cyclohexanehexol ring which can interact with positively charged metal ions and biological molecules [20]. In this study, PA was applied to coat on the LDHs by complexing with antibacterial metal ions without the participation of organic solvent. Hence, as shown in Scheme 1, a green and facile way was proposed to utilize PA-Cu(II) coordination compounds to *in-situ* functionalize LDHs (LDHs@PA-Cu(II)) for the first time. The functional coordination compounds can promote the homogeneous dispersion of LDHs in PLA matrix, contributing to better interface compatibility and interaction between LDHs and PLA. LDHs@PA-Cu(II)/PLA nanocomposite films were fabricated by solution casting evaporation process. The purpose of the present study was to develop biodegradable PLA nanocomposite films with different addition of LDHs@PA-Cu(II) to evaluate microstructural, thermal, crystallization, mechanical, antibacterial, and gas barrier properties for active packaging applications.

## 2. Materials and Methods 

### 2.1. Materials and Chemicals

MgAl-CO_3_ LDHs was synthesized by hydrothermal method according to previous report [17]. Phytic acid (PA) solution (70% in H_2_O), CuCl_2_·2H_2_O were purchased from Shanghai Aladdin Bio-Chem Technology Co., Ltd (China). Poly(lactic acid) (PLA) (4032D, *M*_w_ = 207 kDa) was purchased from Nature Works LLC (Blair, NE, USA). Trichloromethane (analytical purity) was purchased from Xilong Scientific Co., Ltd (Shantou, China).

### 2.2. Preparation of LDHs@PA-Cu(II)

Generally, MgAl-CO_3_ LDHs (0.0500 g) was dispersed in deionized water (100 mL) under ultrasonic treatment for 0.5 h. Furthermore, PA aqueous solution (0.0500 g, 0.053 mmol PA) was added into MgAl-CO_3_ LDHs dispersion. Then, the dispersion was kept magnetic stirring for 15 min to achieve the purpose of adsorption. Subsequently, CuCl_2_·2H_2_O (0.0090 g, 0.053 mmol) was added into the above dispersion under continuous stirring for 2 h. The color of the above dispersion gradually changed to light blue. The product (LDHs@PA-Cu(II)) was obtained by centrifugation, washing and finally drying.

### 2.3. Preparation of LDHs@PA-Cu(II)/PLA Nanocomposites

According to data in Table 1, a certain amount of LDHs@PA-Cu(II) was dispersed in trichloromethane under ultrasonic treatment for 0.5 h. Subsequently, PLA was added to LDHs@PA-Cu(II) dispersion at room temperature under continuous stirring for 4 h. Then, the dispersion was treated with ultrasonic for 15 min. Subsequently, the above dispersion was vaporized to obtain composite films in the horizontal polytetrafluoroethylene molds at room temperature. For comparison, the same processing was used for preparing MgAl-CO_3_ LDHs/PLA nanocomposites (named as LDHs/PLA, MgAl-CO_3_ LDHs loading was 1 wt %).

### 2.4. Characterization

#### 2.4.1. Fourier Transforms Infrared Spectroscopy Analysis

Fourier transform infrared spectroscopy (FT-IR) spectra were recorded by a spectrophotometer (ALPHA, Bruker, Billerica, MA, USA) in the region of 400–4000 cm^−1^ with resolution 4 cm^−1^ for 64 scans. The samples were mixed and ground with KBr and then pressed to pellets. 

#### 2.4.2. X-ray Diffraction Analysis

X-ray diffraction (XRD) analysis was performed on a powder diffractometer (X’pert, Panalytical, Almelo, the Netherlands), using CuKα radiation (1.5406 Å) at a scanning rate of 5 °/min (from 5° to 80°).

#### 2.4.3. Microstructure and Elemental Analysis

The surface micromorphology of the samples was observed by scanning electron microscope (SEM) (sigma500, Zeiss, Oberkochen, Germany) and transmission electron microscope (TEM) (Talos, FEI, Eindhoven, the Netherlands), respectively. For the analysis of fracture surface, the samples were fractured in liquid nitrogen and then coated with gold. Furthermore, chemical elemental analysis of the samples was performed on energy-dispersive X-ray spectrometry (EDS) (X-Max^n^, Oxford, Abingdon, UK).

#### 2.4.4. Differential Scanning Calorimeter Analysis

Thermal analysis was performed on a differential scanning calorimeter (DSC) (DSC214, Netzsch, Selb, Germany). The samples were measured in the temperature range from −30 °C to 220 °C with heating and cooling rates of 10 °C/min using nitrogen as protection gas. The following expression was used to calculate crystallinity degree (χ) of PLA, χ = [∆*H*_m_/(∆*H*_0_ × *φ*)] × 100%, where ∆*H*_m_ and ∆*H*_0_ were the measured melting enthalpy and the melting enthalpy of 100% crystalline PLA (93.7 J/g) [3], and *φ* was the mass fraction of PLA.

#### 2.4.5. Thermal Stability Analysis

Thermal stability analysis was performed on a thermal gravimetric analyzer (TGA) (TG209F3, Netzsch, Selb, Germany). The samples were prepared in alumina pans and heated from 30 to 800 °C in nitrogen atmosphere at a heating rate of 10 °C/min. The mass of the samples was ~10 mg.

#### 2.4.6. Mechanical Property Analysis

Mechanical measurements were carried out on a universal testing machine (ETM502B-Ex, Wance, Shenzhen, China). Mechanical properties were measured at a constant speed of 20 mm/min in general accordance with ISO 527. At least five replicates for each type of the samples were tested.

#### 2.4.7. Oxygen Permeability Analysis

Oxygen permeability was investigated using an oxygen permeability tester (OX-TRAN 2/21, Mocon, Minneapolis, MN, USA) according to ASTM D3985 at 23 °C and 0% RH. Oxygen permeability measurements were conducted on circular films (5 cm^2^).

#### 2.4.8. Antibacterial Activity Analysis

Antibacterial properties of the samples were investigated using shake flask method against *Escherichia coli* [21]. In order to obtain bacteria suspension, *E. coli* was cultured in Luria Broth (LB) solid medium for 24 h at 37 °C. A representative colony was cultured in LB liquid medium, followed by being incubated with shaking for 24 h at 37 °C. Bacterial cell concentration was estimated by measuring the absorbance of bacteria suspension. The bacteria suspension was diluted until the absorbance value of 600 nm was 0.1. The absorbance value was recorded using a UV-visible spectrophotometer (SPECORD 210 PLUS, Analytikjena, Jena, Germany). In total, 150 μL of bacterial solution was dropped into a conical flask containing 15 mL of liquid medium. Films (diameter = 1 cm) and powders (mass = 50 mg), were dipped into falcon tubes containing the above bacterial solution, respectively. After shaking vigorously in a shaking incubator for 24 h at 37 °C, each diluent was spread onto the agar plate. Finally, viable microbial colonies were counted after incubating the plate for 24 h at 37 °C. The unit of value obtained by the method is colony-forming units (CFU)/cm^2^. Furthermore, the antibacterial activity of the samples was evaluated by the antibacterial rate, which was equal to [(control value − sample value)/control value] × 100% [22].

## 3. Results and Discussion

### 3.1. Chemical Structure of MgAl-CO_3_ LDHs and LDHs@PA-Cu(II)

**Figure 1:**
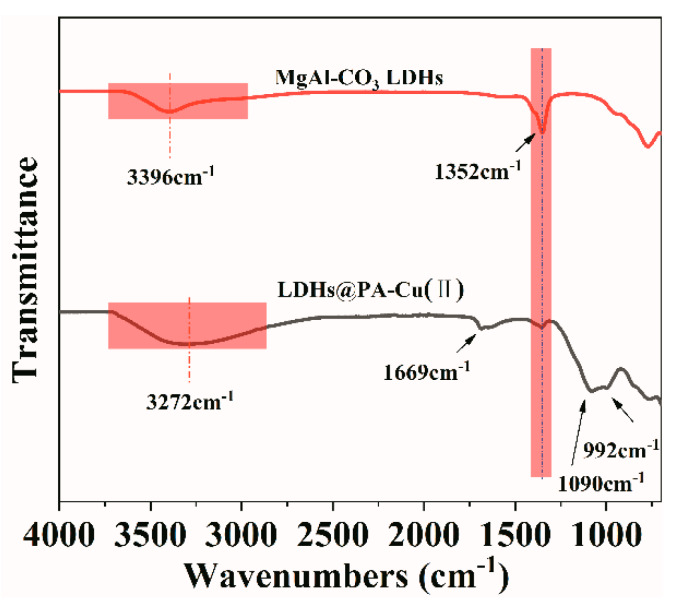
FT-IR spectra of MgAl-CO_3_ LDHs and LDHs@PA-Cu(II).

FT-IR spectra of MgAl-CO_3_ LDHs and LDHs@PA-Cu(II) are shown in Figure 1. FT-IR spectra of MgAl-CO_3_ LDHs and LDHs@PA-Cu(II) show almost identical characteristics of typical LDHs. The infrared absorption peak of MgAl-CO_3_ LDHs at 3396 cm^−1^ is attributed to characteristic stretching vibration of hydroxyl groups and water molecules between layers. Affected strongly by hydroxyl groups of PA, the infrared absorption peak of LDHs@PA-Cu(II) is transferred to 3272 cm^−1^ [23]. Furthermore, the infrared absorption peak at 1352 cm^−1^ for MgAl-CO_3_ LDHs and LDHs@PA-Cu(II) is attributed to the vibrations of CO_3_^2−^ in the interlayer [17]. Compared with MgAl-CO_3_ LDHs, LDHs@PA-Cu(II) shows new peaks at 1669, 1090 and 992 cm^−1^, which attributed to phosphate hydrogen radical (HPO_4_^2−^), phosphate radical (PO_4_^3−^), and P–O–C bonds in PA [20]. In addition, the infrared absorption peaks recorded below 700 cm^−1^ correspond to the vibration of metal-oxygen bonds in the MgAl-CO_3_ LDHs [15]. It is preliminary evidence that PA-Cu(II) coatings are synthesized on the MgAl-CO_3_ LDHs.

### 3.2. Microstructure of MgAl-CO_3_ LDHs and LDHs@PA-Cu(II)

**Figure 2:**
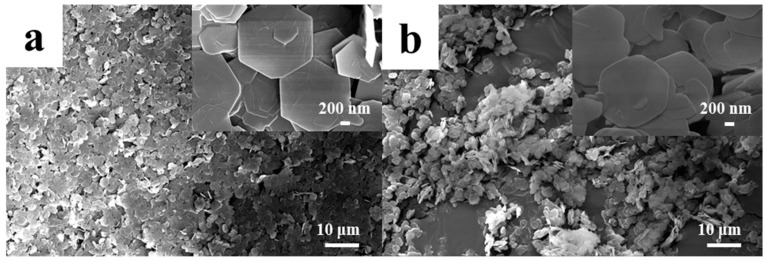
SEM images of (**a**) MgAl-CO_3_ LDHs and (**b**) LDHs@PA-Cu(II) under different magnifications.

SEM images of MgAl-CO_3_ LDHs and LDHs@PA-Cu(II) under different magnifications are shown in Figure 2. The synthetic MgAl-CO_3_ LDHs and LDHs@PA-Cu(II) both clearly display a typical hexagonal layered structure, with a uniform size in ~1500 nm. Meanwhile, compared with MgAl-CO_3_ LDHs, the micromorphology of LDHs@PA-Cu(II) is more rough and mellow (as shown in Figure 2b).

**Figure 3:**
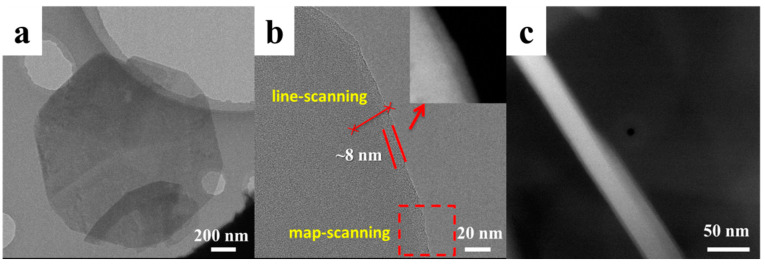
TEM images of (**a**,**b**) LDHs@PA-Cu(II) under different magnifications, and (**c**) MgAl-CO_3_ LDHs from the lateral view.

Upon coating of PA-Cu(II) onto MgAl-CO_3_ LDHs, micromorphology of LDHs@PA-Cu(II) is further analyzed by TEM. As shown in Figure 3, it is revealed that MgAl-CO_3_ LDHs are successfully coated by PA-Cu(II) coordination compounds. Furthermore, the thickness of PA-Cu(II) coatings is ~8 nm. It can be estimated from Figure 3b,c that the thickness of MgAl-CO_3_ LDHs and LDHs@PA-Cu(II) are ~40 nm and ~56 nm, respectively. As a result, the aspect ratio of them are ~37.5 and ~26.8, respectively. The aspect ratio will be used for the calculation of gas relative permeability discussed later [24,25].

**Figure 4:**
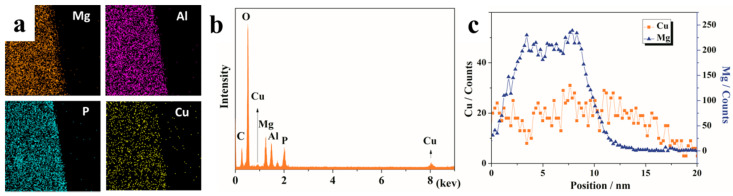
(**a**) EDS map-scanning of LDHs@PA-Cu(II), (**b**) EDS spectrum of LDHs@PA-Cu(II), (**c**) EDS line-scanning of LDHs@PA-Cu(II).

In order to obtain evidence of PA-Cu(II) coordination compounds on the surface of MgAl-CO_3_ LDHs directly, surface chemical elements are also analyzed by EDS (as shown in Figure 4). The related element analysis region is indicated in Figure 3b. In Figure 4a,b, the large peak for phosphorus indicates the existence of PA. The small peaks for copper indicate the existence of Cu(II) [26]. Furthermore, the uniform distribution of these elements reveals the uniformity of PA-Cu(II) coatings. In Figure 4c, EDS line-scanning reveals that the content of magnesium decrease sharply to the edge of LDHs@PA-Cu(II). Instead, the content of copper has little change, which further indicate the presence of PA-Cu(II) coordination compounds.

### 3.3. Antibacterial Properties of LDHs@PA-Cu(II) and LDHs@PA-Cu(II)/PLA Nanocomposites

**Figure 5:**
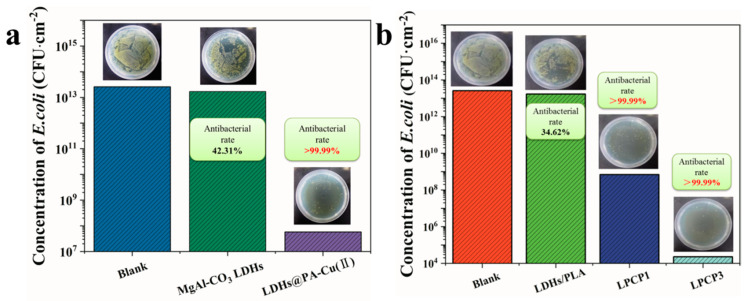
Antibacterial properties of (**a**) LDHs@PA-Cu(II) and (**b**) LDHs@PA-Cu(II)/PLA nanocomposites.

In order to quantitatively evaluate the antibacterial properties of LDHs@PA-Cu(II) and LDHs@PA-Cu(II)/PLA nanocomposites, bacterial colony counter was used to count the number of colonies in the petri dishes with different samples. Furthermore, the antibacterial activity of the samples was evaluated by the antibacterial rate, as shown in Figure 5. In Figure 5, original MgAl-CO_3_ LDHs still shows a certain degree of antibacterial activity, and the antibacterial rate reaches 42.31%. Upon coating of PA-Cu(II) onto MgAl-CO_3_ LDHs, the antibacterial activity of LDHs@PA-Cu(II) is significantly improved, and the antibacterial rate is up to 99.99%. Generally known, copper ion is effectively applied to inhibit bacterial growth in our life and production [27]. However, copper salt used alone is difficult to maintain a sustainable and uniform density of copper ions [27,28]. Therefore, LDHs@PA-Cu(II) can be a novel antibacterial agent to effectively inhibit bacterial growth and solve the problems associated with the direct addition of copper salt. The antibacterial properties of nanocomposites were improved by adding antibacterial nanoparticles. In Figure 5b, not surprisingly, MgAl-CO_3_ LDHs does not improve the antibacterial properties of PLA matrix. Furthermore, the antibacterial rate of LPCP1 and LPCP3 both exceeds 99.99%. With the increase of LDHs@PA-Cu(II), the antibacterial properties of LDHs@PA-Cu(II)/PLA nanocomposites become more and more excellent.

### 3.4. Thermal Stability Properties of LDHs@PA-Cu(II) and LDHs@PA-Cu(II)/PLA Nanocomposites

**Figure 6:**
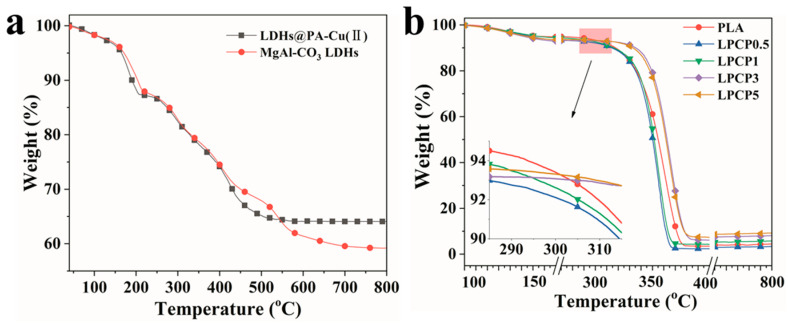
TGA curves of (**a**) LDHs@PA-Cu(II) and (**b**) LDHs@PA-Cu(II)/PLA nanocomposites.

TGA curves of MgAl-CO_3_ LDHs and LDHs@PA-Cu(II) are shown in Figure 6a. MgAl-CO_3_ LDHs and LDHs@PA-Cu(II) exhibit similar two mass loss stages, which can be attributed to thermal decomposition characteristics of typical LDHs in nitrogen atmosphere. For MgAl-CO_3_ LDHs, in the first stage of mass loss, the removal of absorbed water and crystalline water (moisture content ~12%) occur from room temperature to ~215 °C. Furthermore, in the second stage of mass loss, dehydroxylation of lattice and thermal decomposition of carbonate ions in the interlayer occur from ~215–~575 °C [29]. In addition to mass loss characteristics of MgAl-CO_3_ LDHs, mass loss of LDHs@PA-Cu(II) also includes the decomposition of PA-Cu(II) coatings. The results show that the thermal stability of LDHs@PA-Cu(II) is not deteriorated significantly.

TGA curves of LDHs@PA-Cu(II)/PLA nanocomposites are shown in Figure 6b. According to previous research [30,31], the addition of unmodified LDHs or phosphonium-modified LDHs in the PLA matrix leads to an obvious decrease in the thermal stability of PLA matrix. When the addition of LDHs@PA-Cu(II) increases from 0 to 1 wt %, the thermal stability of LDHs@PA-Cu(II)/PLA nanocomposites has the similar trend. Interestingly, this is not yet the case when the addition of LDHs@PA-Cu(II) exceeds 1 wt %. This phenomenon can be explained by the barrier effect of LDHs@PA-Cu(II) when the addition of LDHs@PA-Cu(II) reaches a certain level [32]. LDHs@PA-Cu(II) reduces the initial thermal stability of LDHs@PA-Cu(II)/PLA nanocomposites. However, the overall thermal stability of LDHs@PA-Cu(II)/PLA nanocomposites is improved gradually with the increase of LDHs@PA-Cu(II).

### 3.5. Thermal and Crystallization Properties of LDHs@PA-Cu(II)/PLA Nanocomposites

**Figure 7:**
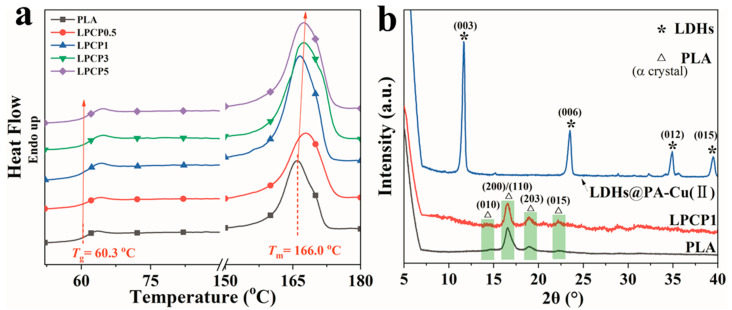
(**a**) DSC second heating curves and (**b**) XRD patterns of LDHs@PA-Cu(II)/PLA nanocomposites and LDHs@PA-Cu(II).

To investigate the effect of LDHs@PA-Cu(II) on the thermal properties of PLA matrix, DSC analysis was performed and DSC second heating curves are shown in Figure 7a. The corresponding data of thermal analysis, such as glass transition temperature (*T*_g_), melting temperature (*T*_m_), melting enthalpy (Δ*H*_m_) and crystallinity (χ), are presented in Table 2. As shown in Figure 7a and Table 2, *T*_g_ of PLA is observed to be 60.3 °C, while that of LDHs@PA-Cu(II)/PLA nanocomposites increases slightly. This can be attributed to stronger interaction of PA-Cu(II) coatings to change the mobility of PLA chains related to the glass transition [33]. The calculated χ of LDHs@PA-Cu(II)/PLA nanocomposites are much higher than that of pure PLA as LDHs@PA-Cu(II) content increases, demonstrating the nucleating effect induced by LDHs@PA-Cu(II). Furthermore, *T*_m_ of LDHs@PA-Cu(II)/PLA nanocomposites show a slight increase compared with that of pure PLA. All the nanocomposites show one melting peak at 166.0~167.5 °C from the second heating scan.

The α-form crystals are exclusively formed in both pure PLA and LDHs@PA-Cu(II)/PLA nanocomposites, as evidenced by the XRD patterns given in Figure 7b. It is noteworthy that the presence of LDHs@PA-Cu(II) has no effect on the crystalline form of PLA. XRD patterns of PLA and LPCP1 exhibit reflections at 2θ = 14.67°, 16.68°, 18.99°, and 22.39°, corresponding to (010), (200)/(110), (203) and (015) planes of the typical orthorhombic crystal form for PLA matrix [33,34]. After the addition of 1 wt % LDHs@PA-Cu(II), the diffraction peak intensity of LPCP1 increased to a certain extent. This is consistent with DSC analysis results. However, there is no visible characteristic diffraction peak (003) of typical LDHs in the LPCP1, which may be due to the low content and diffraction peak intensity of LDHs@PA-Cu(II) [5].

### 3.6. Mechanical Properties and Microstructure of LDHs@PA-Cu(II)/PLA Nanocomposites

**Figure 8:**
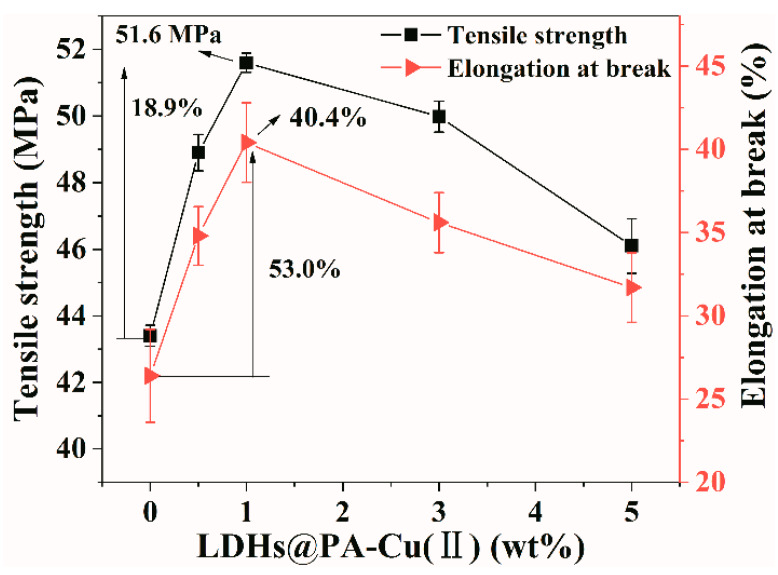
Mechanical properties of LDHs@PA-Cu(II)/PLA nanocomposites.

Mechanical properties of LDHs@PA-Cu(II)/PLA nanocomposites are shown in Figure 8. Compared with pure PLA, LPCP1 shows a 53.0% increase in elongation at break and a 18.9% increase in tensile strength when adding 1 wt % LDHs@PA-Cu(II). According to previous research, the increase of heterogeneous nucleation and interfacial interaction can improve the toughness of material [7]. Furthermore, tensile strength of LDHs@PA-Cu(II)/PLA nanocomposites are continuing to increase when LDHs@PA-Cu(II) content is no more than 1 wt %. The improvement of tensile strength can be attributed to the good adhesion between LDHs@PA-Cu(II) and PLA matrix, which hinders the chains movement and restricts the deformation ability, eventually results in reinforcing effect which increases the resistance to the external mechanical forces [3]. When LDHs@PA-Cu(II) content exceeds 1 wt %, tensile strength and elongation at break of LDHs@PA-Cu(II)/PLA nanocomposites begin to decrease gradually. Nonetheless, when the addition of LDHs@PA-Cu(II) reaches 5 wt %, mechanical properties of LDHs@PA-Cu(II)/PLA nanocomposites is still better than those of pure PLA. As a general rule, except for the change of filler content, the strength of composites also depends on the size and distribution of defects in the composites, and the size and distribution of defects finally determine the loading degree of composites before failure [35]. Good interface interaction can effectively reduce interface defects in the composites. Therefore, it is noteworthy that mechanical properties of PLA can be improved noticeably by adding appropriate amount of LDHs@PA-Cu(II).

**Figure 9:**
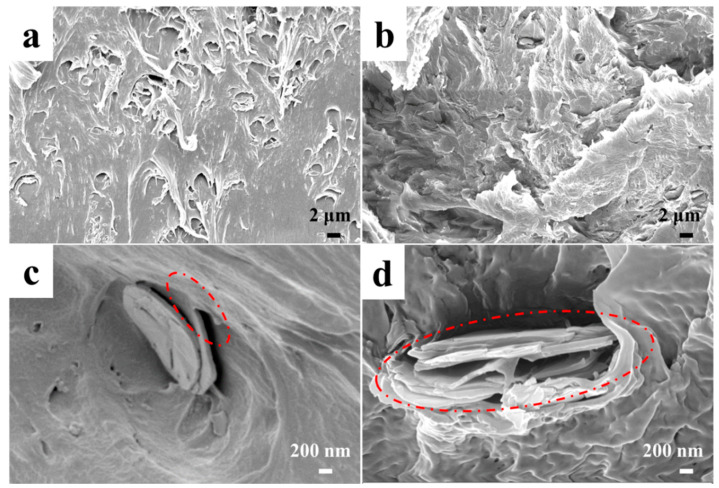
SEM images of fracture surface for LDHs@PA-Cu(II)/PLA nanocomposites with 1 wt % LDHs@PA-Cu(II) (**a**,**c**) and 5 wt % LDHs@PA-Cu(II) (**b**,**d**).

In order to further understand the relationship between interfacial structure and mechanical properties, SEM was employed to analyze the fracture surface of LDHs@PA-Cu(II)/PLA nanocomposites, as shown in Figure 9. According to previous research [5,36], overall fracture surface of pure PLA is rough, which indicates plastic deformation. Figure 9a,b both show rough and well stretched surface (a large number of curled broken fibrils), indicating LDHs@PA-Cu(II) can improve the flexibility of PLA matrix [37,38,39]. As shown in Figure 9c, LDHs@PA-Cu(II) tightly combines with PLA matrix without visible interface gap. Furthermore, LDHs@PA-Cu(II) (1 wt %) is uniformly distributed in PLA matrix with a small amount of aggregates. Generally, relatively good dispersion of nanoparticles can be achieved in the nanocomposites with low nanoparticles content [5]. In Figure 9d, apparently, LDHs@PA-Cu(II) (5 wt %) shows much more heterogeneous dispersion with apparent agglomerates, which indicates that the interfacial compatibility and dispersibility of LDHs@PA-Cu(II) get worse [6]. To some extent, this can explain the reasons for the change of mechanical properties with LDHs@PA-Cu(II) contents increase. Meanwhile, the better interfacial bonding, the fewer the interfacial defects, which is also closely related to the better gas barrier properties of materials [17].

### 3.7. Oxygen Barrier Properties and Mechanism of LDHs@PA-Cu(II)/PLA Nanocomposites

**Figure 10:**
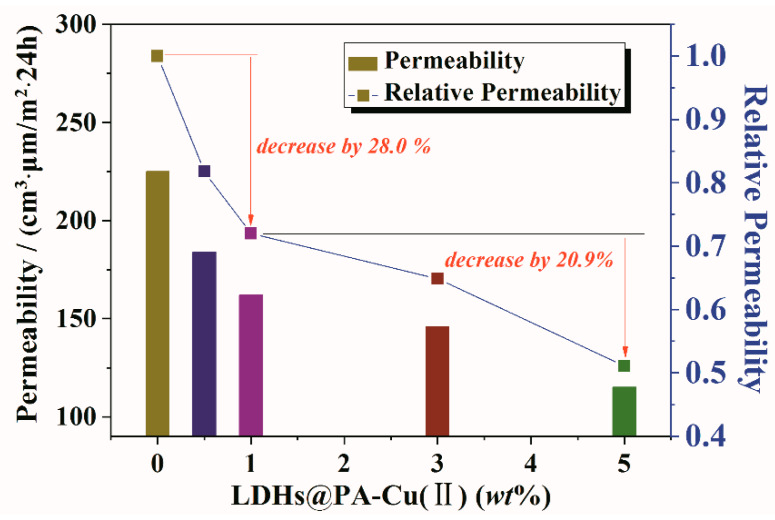
O_2_ permeability of LDHs@PA-Cu(II)/PLA nanocomposites and pure PLA.

Oxygen permeability of LDHs@PA-Cu(II)/PLA nanocomposites is shown in Figure 10. When LDHs@PA-Cu(II) content reaches 1 wt %, *R*_p_ (relative permeability = P/P_0_) is decreased obviously to 72.0%, thereafter it slowly decreases. Only when LDHs@PA-Cu(II) is better dispersed in PLA matrix and the interface is stronger between clay and PLA matrix can this be achieved, which contributes to the reduced interface defects and tortuous path of gas diffusion [40,41]. *R*_p_ of LDHs@PA-Cu(II)/PLA nanocomposites decreases by only 20.9% as LDHs@PA-Cu(II) content increases from 1 wt % to 5 wt %. This reflects a significant reduction in barrier efficiency of LDHs@PA-Cu(II). Therefore, PA-Cu(II) coatings play an important role in reducing gas permeability in LDHs@PA-Cu(II)/PLA nanocomposites.

According to considerable reports in the literature [42,43,44], the barrier mechanism of layered clay is mainly related to the bulk effect and barrier effect. As a rule, layered clay/polymer nanocomposites are composed of a permeable phase (polymer matrix) and an impermeable phase (layered clay). The bulk effect of layered clay can hinder the dissolution of gas molecules in the composite films. The barrier effect of layered clay can prevent permeation of the gas molecules through the composite films. 

Nielsen’s detour theory was first proposed, and has been used to predict the *R*_p_ of filled polymer composites. So far, many permeability prediction models are still optimized based on this theory [45]. Based on Nielsen’s detour theory, classical relative permeability equation can be expressed as follows,

(1) $$R_{p}=\frac{\left(1-\emptyset_{s}\right)}{1+\frac{L}{2W}\emptyset_{s}}$$

where *L*, *W*, and *ϕ*_s_ represent the volume fraction, diameter and thickness of LDHs@PA-Cu(II), respectively. The aspect ratio (*L*/*W*, ~26.8) has been calculated in the previous analysis. 

**Figure 11:**
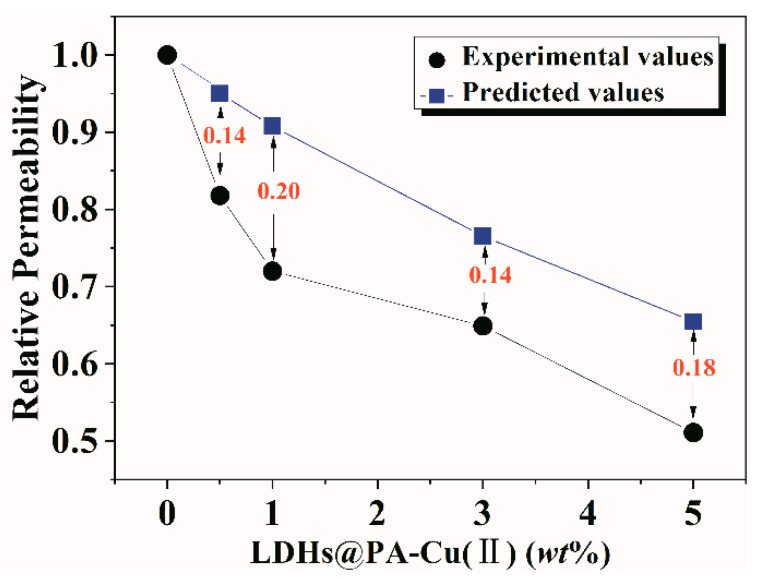
Relative permeability for experimental and predicted values.

According to the relative permeability equation, experimental values and the predicted of *R*_p_ can be calculated as shown in Figure 11. The results show that *R*_p_ of the experimental values is significantly lower than that of the predicted values. Furthermore, the deviation (0.14~0.20) between experimental and predicted values is large in the whole region. The reason is that Nielsen’s model is based on geometric analysis entirely. Two important factors are not considered in this model: the effect of layered clay on the structure of polymer matrix (such as crystallization) and the interface interaction [45]. The interfacial incompatibility can lead to the formation of interface defects and eventually the increase of gas permeability. Considering our layered clay/polymer nanocomposites, the introduction of LDHs@PA-Cu(II) can cause interaction of different types toward PLA matrix, resulting in more strong interaction interface [40,46].

## 4. Conclusions

LDHs@PA-Cu(II) was firstly synthetized via facile deposition and chelation of PA-Cu(II) coordination compounds on the surface of LDHs. Furthermore, LDHs@PA-Cu(II)/PLA nanocomposites were prepared by blending LDHs@PA-Cu(II) and pure PLA via solution casting evaporation process. It is revealed that PA-Cu(II) coordination compounds are uniformly coated on MgAl-CO_3_ LDHs at a thickness of ~8 nm. With this nanoscale coatings, the mechanical, and gas barrier properties of LDHs@PA-Cu(II)/PLA nanocomposites are improved significantly, which are attributed to strong interface interaction and heterogenous nucleation. After adding only 1 wt % LDHs@PA-Cu(II), elongation at break and tensile strength increase by 53.0% and 18.9% respectively, and the oxygen relative permeability decreases by 28.0%. However, excess addition of LDHs@PA-Cu(II) lead to the decrease of mechanical properties. Nonetheless, elongation at break and tensile strength of LDHs@PA-Cu(II)/PLA nanocomposites are both higher than those of pure PLA. The analysis of barrier mechanism reveals that the important factors for improving the barrier properties are not only the bulk effect and barrier effect of LDHs@PA-Cu(II), but also the specific interactions between LDHs@PA-Cu(II) and PLA matrix. Meanwhile, due to the antibacterial activity of PA-Cu(II) coatings, the antibacterial rate of LDHs@PA-Cu(II) exceeds 99.99%. Furthermore, the corresponding LDHs@PA-Cu(II)/PLA nanocomposites also shows outstanding antibacterial properties. Therefore, antibacterial LDHs@PA-Cu(II)/PLA nanocomposites have broad application prospects in active packaging due to their biodegradability and physical properties.

## Figures and Tables

**Table 1:** Composition of LDHs@PA-Cu(II)/PLA nanocomposites.

Sample	PLA/g	LDHs@PA-Cu(II)/g	LDHs@PA-Cu(II)/wt %
PLA	1.4	0	0
LPCP0.5	1.4	0.0070	0.5
LPCP1	1.4	0.0140	1
LPCP3	1.4	0.0433	3
LPCP5	1.4	0.0737	5

**Table 2:** The related results of LDHs@PA-Cu(II)/PLA nanocomposites according to thermal analysis.

Sample	*T*_g_/°C	*T*_m_/°C	Δ*H*/J·g^−1^	χ/%
PLA	60.3	166.0	32.31	34.5
LPCP0.5	60.9	167.8	36.59	39.2
LPCP1	60.5	166.6	50.83	54.8
LPCP3	61.2	167.4	48.45	53.3
LPCP5	60.9	167.5	45.91	51.6
